# Integrated Efforts for the Valorization of Sweet Potato By-Products within a Circular Economy Concept: Biocomposites for Packaging Applications Close the Loop

**DOI:** 10.3390/polym13071048

**Published:** 2021-03-27

**Authors:** Micaela Vannini, Paola Marchese, Laura Sisti, Andrea Saccani, Taihua Mu, Hongnan Sun, Annamaria Celli

**Affiliations:** 1 Department of Civil, Chemical, Environmental, and Materials Engineering, University of Bologna, Via Terracini 28, 40131 Bologna, Italy; paola.marchese@unibo.it (P.M.); laura.sisti@unibo.it (L.S.); andrea.saccani@unibo.it (A.S.); annamaria.celli@unibo.it (A.C.); 2 Laboratory of Food Chemistry and Nutrition Science, Institute of Food Science and Technology, Chinese Academy of Agricultural Sciences; Key Laboratory of Agro-Products Processing, Ministry of Agriculture and Rural Affairs, No. 2 Yuan Ming Yuan West Road, Haidian District, Beijing 100193, China; mutaihua@126.com (T.M.); honey0329@163.com (H.S.)

**Keywords:** bio-based polymers, sweet potato residues, biocomposites, natural fillers, sustainability

## Abstract

With the aim to fully exploit the by-products obtained after the industrial extraction of starch from sweet potatoes, a cascading approach was developed to extract high-value molecules, such as proteins and pectins, and to valorize the solid fraction, rich in starch and fibrous components. This fraction was used to prepare new biocomposites designed for food packaging applications. The sweet potato residue was added to poly(3-hydroxybutyrate-*co*-3-hydroxyvalerate) in various amounts up to 40 wt % by melt mixing, without any previous treatment. The composites are semicrystalline materials, characterized by thermal stability up to 260 °C. For the composites containing up to 10 wt % of residue, the tensile strength remains over 30 MPa and the strain stays over 3.2%. A homogeneous dispersion of the sweet potato waste into the bio-polymeric matrix was achieved but, despite the presence of hydrogen bond interactions between the components, a poor interfacial adhesion was detected. Considering the significant percentage of sweet potato waste used, the biocomposites obtained show a low economic and environmental impact, resulting in an interesting bio-alternative to the materials commonly used in the packaging industry. Thus, according to the principles of a circular economy, the preparation of the biocomposites closes the loop of the complete valorization of sweet potato products and by-products.

## 1. Introduction

The principles of a circular economy are based on the consideration that waste and pollution are precious resources of added-value molecules, that can be exploited to obtain new ingredients and materials, which can, at the end of their life, return to the ground [1]. More specifically, in the agricultural sector, food production leads to large amounts of waste that correspond to 140 billion tons of biomass, generated globally every year [2]. Interestingly, these wastes are rich in valuable substances that can be further exploitable [3,4,5,6]; In addition to that, even the remaining residues after the extraction of all the precious substances can be valorized by finding a profitable way as an alternative to landfill or incineration [7,8]. This integrated cascading approach makes it possible to design a zero-waste paradigm. 

A typical example of a food for which this concept is relevant is sweet potato (*Ipomoea batatas* Lam.): its processing for food purposes produces a large amount of waste that cannot be ignored. Indeed, the production processes of sweet potato starch generate sweet potato residues and wastewater. In 2019, in China—the largest producer of sweet potatoes in the world—the yield of fresh sweet potato alone exceeded 52 million tonnes [9]. Meanwhile, approximately 4.5–5.0 tonnes of fresh sweet potato residues are generated for every tonne of produced starch. However, while a small amount of the sweet potato residue by-products is used as low-value animal feed, most of it is thrown away, and thus becomes a major element of environmental pollution. Mei et al. [10] reported that sweet potato residues consist of starch (51.98%), dietary fibre (21.41%) and protein (4.55%) (dry basis). Moreover, the average content of cellulose, lignin, pectin and hemicellulose are 31.19%, 16.85%, 15.65% and 11.38% (dry basis) in sweet potato dietary fibre, respectively. Therefore, these wastes can be further valorized in many different ways, such as the production of hydrogen [11], lipopeptides and poly-γ-glutamic acid [12], cellulose nanocrystals [13], food material such as residual flour, soluble dietary fibre and pectin [14,15,16], solid emulsifiers [17] etc. Interestingly, potato wastes have also been used as a carbon source in the microorganism fermentation for the synthesis of bio-polyesters: it is reported that microorganisms, as *Cupriavidus necator* [18], *Alcaligenes eutrophus* [19], *Bacillus cereus* or *Bacillus thuringiensis* [20] produced polyhydroxyalkanoates (PHA) from potato waste.

The polyhydroxyalkanoates are currently very attractive materials as they can be derived from biomass and even from the wastes remaining after food processing. In addition to that, they are biodegradable. 

Poly(3-hydroxybutyrate) (PHB), a homopolyester consisting merely of a 3-hydroxybutyrate building block, is the first discovered member of the PHA family. This material features a rather high crystallinity and limited processability, but these issues can be overcome by interrupting the crystalline PHB macromolecular structure by adding building blocks like 3-hydroxyvalerate, resulting in co-polyesters with enhanced material properties and a broader range of applications. In fact, poly(3-hydroxybutyrate-*co*-3-hydroxyvalerate) (PHBV), containing hydroxyvalerate units, is less brittle than PHB and its gas barrier properties to oxygen and aroma are close to those of the polyethylene terephthalate (PET) [21]. In particular, among the biodegradable polymers, PHB and PHBV are the most performant polymers in terms of gas barrier properties: they have the lowest water and oxygen permeability [22]. For this reason, the predictable application of these biomaterials lies in food packaging, even more so as they are approved by the FDA for food contact. Moreover, recent studies report the possibility to further improve their barrier performances by adding organic fillers or bio-derived polymers like nanocellulose [23,24,25,26]. 

However, in general, they feature poor mechanical properties and their production costs are currently around 5 €/kg, higher than fossil-based commercial polyesters. One common way to overcome these drawbacks is to mix these biopolymers with suitable additives or fillers to obtain biocomposites. Some reports in the literature attempt to enhance the physical and mechanical properties of PHBV or other biopolyesters by blending or filling techniques, using different types of natural fibres [21,27,28,29,30,31,32,33] or wastes from agro-industrial processing [34,35,36,37,38,39,40,41]. 

**Figure 1:**
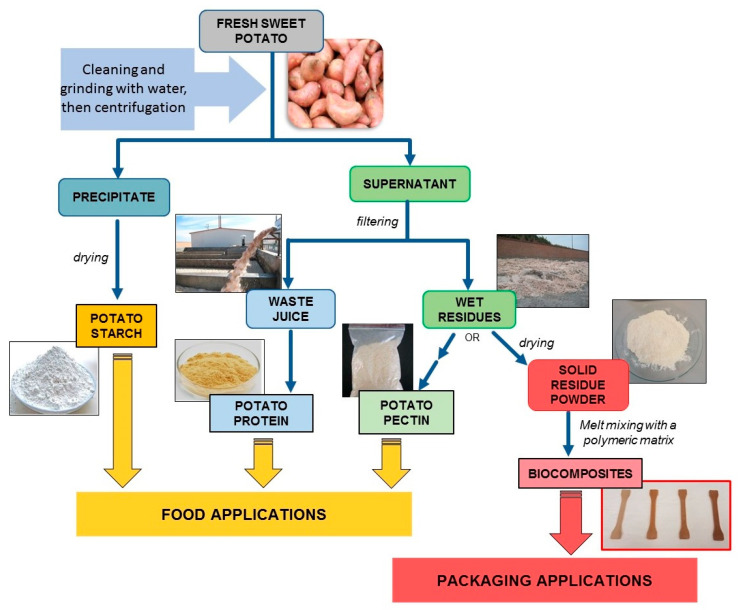
Cascading approach developed in the framework of the NoAW project to valorize the by-products obtained after the extraction of starch from potatoes.

Within the framework of the European project H2020 NoAW (No Agricultural Waste Innovative approaches to turn agricultural waste into ecological and economic assets) [42], a cascading approach to valorize the by-products obtained from the industrial process of starch extraction from potatoes has been developed, as shown in Figure 1, according to the challenging vision for the management of food waste described in Gontard et al. [43]. 

More specifically, potato fruit juice, an aqueous by-product of the potato starch industry, containing 2–5% dry matter, of which one-third is protein, peptides and amino acids/amines, was used to extract proteins. Two different traditional methods (isoelectric precipitation and ammonium sulfate precipitation) [44,45] to recover proteins were applied in order to supplement the limited information available on the comparative results obtainable and to provide some theoretical basis for the recovery and application of potato protein concentrates. 

Moreover, potato pulp is rich in pectin, which can be used as a good raw material for pectin extraction. The acid extraction method is often used in the food industry to extract pectin because of its convenient and easy operation [46].

Finally, the present work also considers, for the first time, the possibility to valorize the solid residue remaining after the starch extraction from Chinese sweet potatoes, by using it as a filler in PHBV-based biocomposites. The use of this type of residue guarantees the biodegradability retention of the composite, as already demonstrated for both the starch and the PHA/starch blends [47,48]. The new exploitation of sweet potato residues in the materials sector once again proves that for every residue a valorization route can be found.

Moreover, particular attention was devoted to the production of composites, which should be obtained by eco-sustainable procedures. Therefore, in the present study, chemical or physical treatments related to the matrix or sweet potato residue [49] have been excluded. No organic or inorganic additives favouring processability or compatibilization between the different phases have been used, in order to limit process costs and any environmental impact.

Therefore, the biocomposites examined in this work can be considered as eco-sustainable, biodegradable and completely derivable from sweet potato wastes, in terms of both the polymeric matrix and the used filler, perfectly meeting the demand for closed-loop materials from the bio-circular economy.

## 2. Materials and Methods

### 2.1. Materials

The sweet potato residues were obtained from the cultivar Shangshu No. 19, one of the most common cultivar for starch extraction. In particular, sweet potatoes were harvested in the year 2016 in the Sweet Potato Research Institute, Chinese Academy of Agricultural Sciences in Jiangsu Province, China. 

A commercial polyhydroxyalkanoate, PHI 002, supplied from NaturePlast (France), was used as a matrix for composite preparation. PHI 002 is a poly(3-hydroxybutyrate-*co*-3-hydroxyvalerate) (in text PHBV) copolyester containing 2 mol % of a hydroxyvaleric unit and 98 mol % of a hydroxybutyric unit (as determined by ^1^H NMR analysis), characterized by a density of 1.23 g/cm^3^ and a melt flow index (190 °C, 2.16 kg) of 5–10 g/10 min.

### 2.2. Production of Sweet Potato Residues

Sweet potato residues resulted from the starch extraction process, which was performed via a following slightly modified Deng method [50]: fresh sweet potato roots were washed, placed in tap water (1:2, *w*/*v*) and then ground with a HR1861/30 juice extractor (Philips, Beijing, China). The resulting slurry was centrifuged at 1875 rpm for 10 min with LXJ-IIC centrifuge (Shanghai, China): the settled solid, consisting of sweet potato starch was collected, separately from the supernatant clear yellowish filtrate, known to be comparable to industrial sweet potato fruit juice. Finally, the sweet potato residue, remaining after the extraction process, was hot air dried at 70 °C for 24 h, then ground into flour by a high-speed universal pulverizer (FW100, Tianjin, China) and kept in zip-lock bags and stored in a dark place at room temperature for further analysis and processing, object of this work.

### 2.3. Composition of Sweet Potato Residues

The starch, protein, ash, fat and dietary fibre contents were determined according to Association of Official Analytical Chemists (AOAC) methods [51]. Starch in the residue sample was firstly hydrolyzed into glucose using thermo-stable α-amylase (A3306, Sigma, St. Louis, MO, USA) and amyloglucosidase (A9913, Sigma, St. Louis, MO, USA). Then the amount of glucose was determined with a glucose assay kit (GAG020, Sigma, St. Louis, MO, USA), and the starch content was calculated as glucose × 0.9 (AOAC method 996.11). Protein was determined as total nitrogen content according to the Kjeldahl procedure, and a factor of 6.25 was used for the conversion of nitrogen to crude protein (method 955.04). Ash was prepared by incinerating samples in a muffle furnace at 550 °C for 2 h (AOAC method 923.03) [51]. The amount of fat was determined using the AOAC method 960.39 [51]. 

Dietary fibres were assessed through the AOAC method 991.43 [51]. Briefly, the residue sample was treated with thermo-stable α-amylase, and then digested with protease (P3910, Sigma, St. Louis, MO, USA), followed by incubation with amyloglucosidase to remove starch and protein components. Insoluble dietary fibre was obtained by centrifugation at 1875 rpm for 15 min at 25 °C, and the soluble dietary fibre was precipitated in 95% ethanol. Total dietary fibre was calculated as the sum of soluble and insoluble dietary fibre.

Cellulose, pectin, lignin and hemicellulose contents were determined by the method reported by Mei et al. [10]. Briefly, the dietary fibre sample was treated with 0.2 M phosphate buffer solution (pH 7.0) at the ratio of 1:10 (*w*/*v*) for 2 h at 20 °C and then centrifuged at 1875 rpm for 15 min. The procedure was repeated three times and the supernatant was collected. The precipitate was extracted with 0.01M EDTA solution for 2 h to bind cations and solubilize pectic substances, the extracted mixtures were filtered by vacuum filtration with a microfiltration membrane (pores = 0.8 μm), filtrates were collected, and the extraction was repeated twice. The supernatants and filtrates were dialyzed and freeze dried to obtain soluble pectic substances. After the extraction of the soluble pectic substances with EDTA, the remaining residues were washed twice with 80% ethanol and three times with distilled deionized water to remove the ethanol. Then the residues were freeze dried for further analysis. The freeze dried sample was treated with thermo-stable α-amylase, amyloglucosidase and protease, successively, to eliminate starch and protein. Finally, the mixture was filtered by vacuum filtration with a microfiltration membrane (pores = 0.8 μm); residues were washed three times with 80% ethanol, once with 95% ethanol and three times with distilled deionized water and then freeze-dried. The residues obtained were extracted three times using 0.5% (*w*/*v*) ammonium oxalate solution at 85 °C for 2 h. The fibre residue was filtered and washed with ethanol and distilled water and then freeze-dried. Filtrates were collected, dialyzed and freeze dried, and insoluble pectic substances were obtained. The sum of soluble and insoluble pectic substances amounted to the pectin content in sweet potato dietary fibre. The fibre residues resulting from insoluble pectic substances were used to fractionate hemicellulose, cellulose and lignin according to the method described by Claye et al. [52]. Then the contents of hemicellulose, cellulose, and lignin in sweet potato dietary fibre were calculated.

### 2.4. Composite Preparation

Both sweet potato residues and commercial PHBV were dried under vacuum at 60 °C overnight. Then, to get composites, the residue and the polymer were melt mixed in a Brabender microcompounder (Milan, Italy), feeding 45–50 g of charge and setting the screw speed at 50 rpm. The blending was carried out at two different processing temperatures and times: at 200 °C for 5 min or at 180 °C for 6 min. The composites were prepared with an amount of filler in the range of 5–40% by weight. 

Composite films were obtained by compression moulding: 3.20 g of ground compounds were scattered on a Teflon foil (12 × 12 cm) within an aluminium frame (10 × 10 cm), 300 µm thick and then covered by a second Teflon foil; the resulting foils couple was placed between the plates of a Carver press (Wabash, IN, USA), then heated at 190 °C under 4.5 bars for 30 s. The films were quickly cooled to room temperature, later led to room pressure, and finally separated from the Teflon foils.

### 2.5. Filler and Composite Characterizations

The pristine PHBV and the composites obtained were analyzed by ^1^H NMR, using a Varian Mercury 400 spectrometer (Palo Alto, CA, USA). Chemical shifts are downfield from tetramethylsilane and a mixture of CDCl_3_/trifluoroacetic acid, 80/20 (*v*/*v*) is used as a solvent. The spectra were acquired just after dissolution in order to avoid the esterification reaction of end groups with trifluoroacetic acid. 

A PerkinElmer Spectrum One FT-IR spectrometer (Milan, Italy) equipped with a Universal ATR sampling accessory instrument was used to collect the spectra and 64 scans at a resolution of 2 cm^−1^ were averaged in a range between 4000 and 700 cm^−1^. 

To determine molecular weights, the composite samples were dissolved in a mixture of CHCl_3_/1,1,1,3,3,3-hexafluoro-2-propanol (HFIP) 95/5 (*v*/*v*) and filtered through a Teflon syringe filter with a pore size of 0.45 mm to eliminate the insoluble fraction. Then, gel permeation chromatography (GPC) measurements were performed at 30 °C on Hewlett Packard Series 1100 liquid chromatography (Milan, Italy) using a PL gel 5 mm Minimixed-C column with chloroform as eluent with a 0.3 mL/min flow; a Refractive Index detector was used and a calibration plot was constructed with monodisperse polystyrene standards. 

Thermogravimetric analysis (TGA) was performed using a PerkinElmer TGA4000 apparatus (Milan, Italy) in nitrogen (gas flow: 40 mL/min) at 10 °C/min heating rate, from 25 to 700 °C. The degradation temperature (*T*_D_) was calculated as the temperature of the maximum degradation rate; the onset degradation temperature (*T*_onset_) was defined as the intercept of the tangent drawn at the inflection point of the decomposition step with the horizontal zero-line of the thermogravimetric curve. 

Calorimetric analysis (DSC) was carried out by means of a PerkinElmer DSC6 calorimeter (Milan, Italy), calibrated with high-purity standards. Thermal treatments were performed under a nitrogen flow as follows: first scan, from −30 to 210 °C at 20 °C/min and 1 min of isotherm at 210 °C; cooling scan, from 210 to −30 °C at 20 °C/min and 1 min of isotherm; second scan, from −30 to 210 °C at 20 °C/min. 

The tensile properties of composites were determined on dumbbell-shaped specimens (2 × 5 × 30 mm) obtained by injection moulding (MegaTech Tecnica DueBi injection moulding machine, Ancona, Italy), working at 165 °C. The tests were carried out on five samples for each composition by an INSTRON 5966 dynamometer (Turin, Italy) equipped with a 10 kN load cell (test speed 5 mm/min, room temperature 19 ± 1 °C and 70 ± 10% of relative humidity. The value of the Young modulus was obtained from the stress/strain curve without using an extensometer, thus providing a value lower than the one usually reported for PHBV.

X-ray diffraction (XRD) patterns of both sweet potato residue powder and PHBV composite films were collected at room temperature in the 5.0–60.0° 2θ interval using a PANalytical X’Pert Pro diffractometer equipped with a copper radiation (λ = 0.15418 nm) and a fast solid state X’Celerator detector. 

To determine the surface hydrophobicity of the composite films, water contact angle measurements were performed using a drop shape analyzer (DSA30S- Kruss, Hamburg, Germany). The drop (4 μL) of distilled water was positioned on the surface of the film by an auto-syringe. Images were captured 5 s after a drop of distilled water was deposited on the surface of the composite films. The reported contact angle values are the mean value of at least eight different measurements. 

In order to investigate the morphology of sweet potato residues and the microstructure of the samples submitted to mechanical tests, a FEI XL20 scanning electron microscopy (SEM, Philips, Milan, Italy) was used. Prior to all observations, specimens were in a vacuum sputtered with gold or aluminium. A 10 kV tension was applied during the analysis.

## 3. Results and Discussion

### 3.1. Characterization of Sweet Potato Processing Residues

The process of starch extraction from sweet potatoes produces two different wastes: the potato fruit juice, particularly rich in protein, and the solid residue, which mainly consists of starch and dietary fibre, as reported in Table 1. Among sweet potato dietary fibre, more than 60% of the composition is insoluble (Table 2).

Therefore, the residue was composed mainly of starch and cellulose, with a relatively low hydrophobic lignin content, meaning a generally hydrophilic character of the residue. This waste differs from the potato pulp used by Righetti and co-workers [34] in a few aspects: the starting crop and a more than doubled starch content. At the same time, the presence of lignin, pectin and protein distinguishes sweet potato residue from simple starch, making it an interesting waste, worthy of extensive investigations.

**Figure 2:**
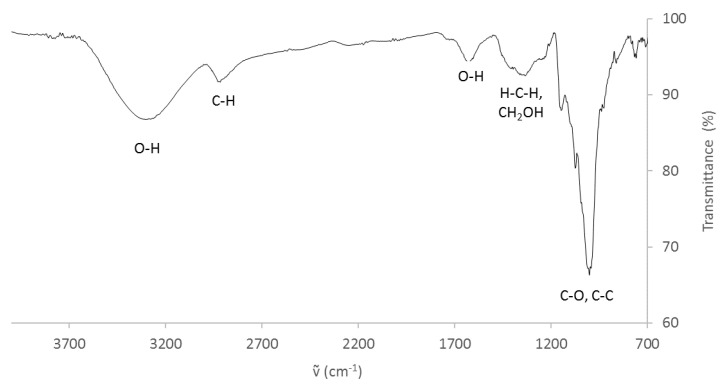
ATR FT-IR of sweet potato residue.

The residue composition was confirmed by FT-IR analysis, as shown in Figure 2. Overall, the typical band profile produced by sweet potato starch is recognizable: absorption bands at 3302 cm^−1^ due to O-H stretching and at 2930 cm^−1^ produced by the stretching of the C–H bond are observable [53]. Moreover, the band related to the bending vibration of water, included in the starch matrix, is visible at 1624 cm^−1^, as well as the vibrations due to symmetric deformation bonding of HCH and CH_2_OH at 1409 and 1336 cm^−1^ and finally the C–O and C–C bond elongations, absorbing between 1150 and 900 cm^−1^. 

**Figure 3:**
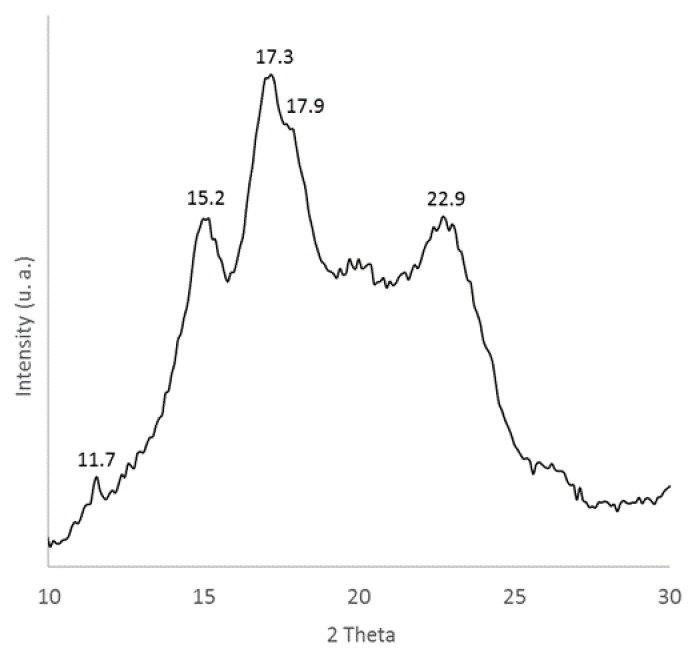
X-ray diffractogram of sweet potato residue.

Sweet potato residue was further characterized by X-ray diffraction and SEM analyses to evaluate the structure and the morphology of the filler. The diffractogram reported in Figure 3 shows that starch, the main component of sweet potato residue, is a semicrystalline polymer. In particular, the presence of the reflections at 15.2°, 17.3°, 17.9°, 22.9° (2θ) is ascribed to the A-type crystalline structure [50,54]. Starch can be classified into three crystalline forms: A, B and C, which is a mixture of A and B-types. Both orthorhombic A and hexagonal B-type starches are based on parallel double helices, but in the A-type the helices are more closely packed whereas the lattices of B-type starches have larger channels in which water molecules can be included [55]. The denser A-type crystalline phase is usually associated with a high amount of amylopectin fraction and it is the typical phase of sweet potato starch [50,56]. Instead, potato starch is commonly characterized by a B-type structure, richer in amylose amorphous fraction.

**Figure 4:**
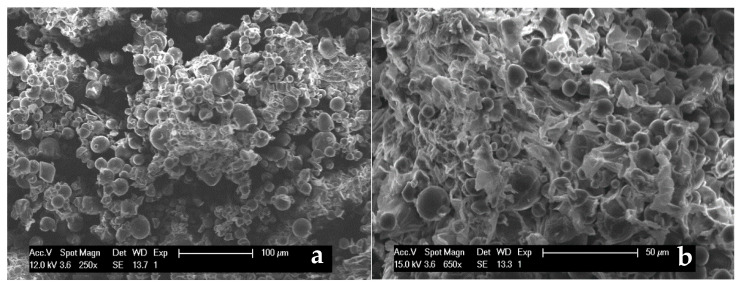

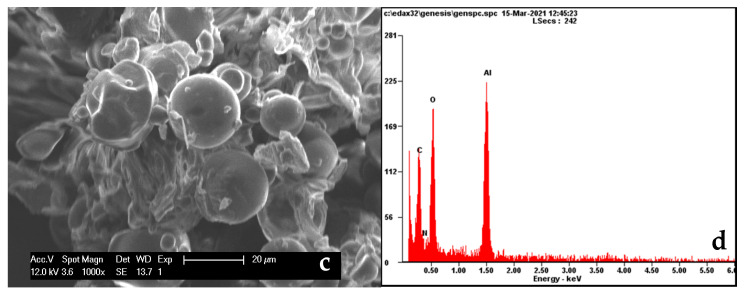
SEM pictures (**a**–**c**) of the sweet potato residue at (**a**) magnification 250×, (**b**) magnification 650×, (**c**) magnification 1000× and EDX spectrum (**d**) of the sweet potato residue metallized by aluminium.

The three-dimensional structure of the examined sweet potato residue consists of an array of individual starch granules, as shown in Figure 4a–c, while the EDX spectrum is displayed in Figure 4d. The roundish structure of the particles corresponds to the one described by Gallant [57], whereas the particle size can vary with different sweet potato strains and physical treatments: in our case, it is included in the range 4–20 μm. The individual starch grains are enveloped and held together by a flat-shaped phase.

**Figure 5:**
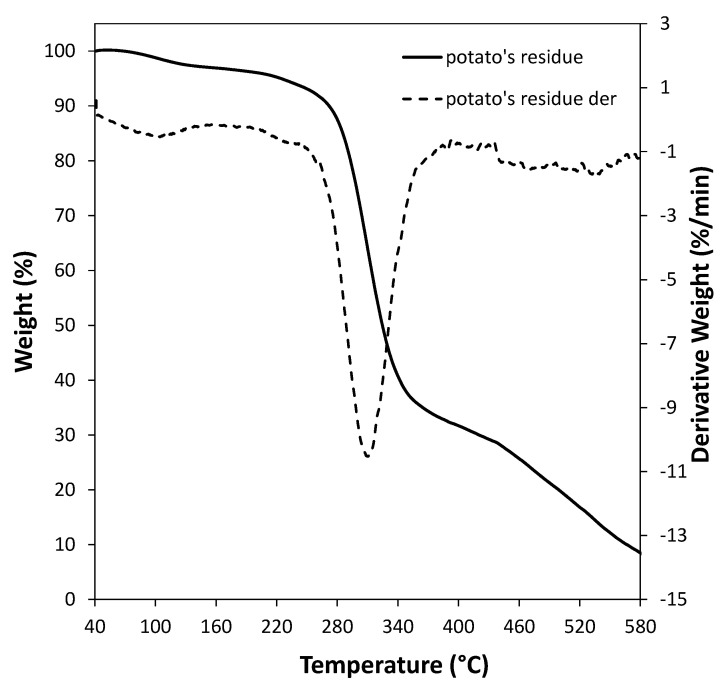
Thermogravimetric analysis (TGA) curve (solid line, left axis) and related derivative curve (dotted line, right axis) of sweet potato residue.

Finally, the residue was characterized through a TGA analysis. Figure 5 shows that, after the initial moisture loss, the residue degradation occurs in a main single step in the temperature range of 250–400 °C, corresponding to the concurrent degradation of cellulose, hemicellulose, starch, pectin and lignin. It is reported that starch decomposes between 250–500 °C [58] and pectin degrades in the region from 190 to 400 °C [59], while the decomposition of hemicelluloses and cellulose occurs from 200 to 335 °C and 335–380 °C, respectively, and, finally, the lignin degrades between 230 and 500 °C [23].

### 3.2. Characterization of PHBV-Based Composites

Considering the high thermal stability of sweet potato residue and the melting process of PHBV, which takes place in the 140–180 °C temperature range, two different mixing conditions for biocomposite preparation were used: 6 min at 180 or 5 min at 200 °C. The filler was actually added into the Brabender chamber only after the complete melting of the polymeric matrix, which required 2 min at 200 °C and 3 min at 180 °C.

Five bio-composites were prepared by adding from 5 to 40 wt % of sweet potato residue to the PHBV matrix. The addition of the filler into the polymeric matrix was performed by simply mixing the components into the molten polymer. This method is green and timesaving since it needs no solvents and only requires a few minutes of mixing. Moreover, no physical or thermal treatments have been previously applied to the components to promote filler-polymer compatibility. 

**Figure 6:**
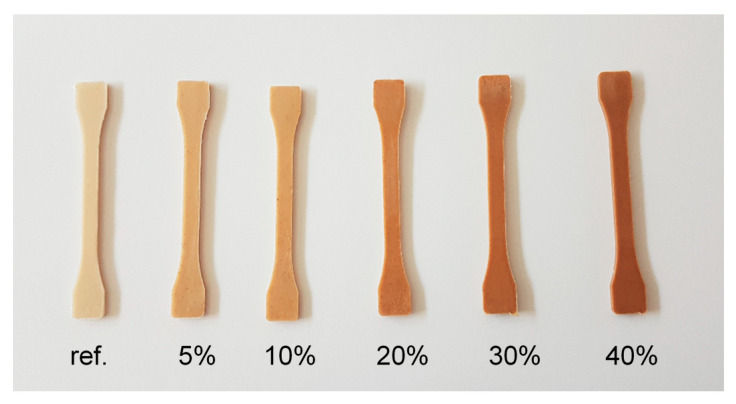
Biocomposite aspects with increasing filler content (from 0 to 40 wt %).

The growing amount of sweet potato residue imparts an increasing brown tone to the original colour of the PHBV. This effect is represented in Figure 6, where dog bones obtained from the different formulations are shown.

The GPC analyses performed on PHBV molten in the Brabender mixer show that the mixing temperature has a relevant effect on the polymeric chain length: although 200 °C is a temperature lower than the initial degradation temperature of PHBV, the polymer seems to suffer under thermal processing. Indeed, as evidenced in Table 3, the mixing at 200 °C leads to a macromolecular weight halving, while for 180 °C this decrement is limited to about 1/3. 

Then, a methodical analysis of the thermal processing effect was conducted, comparing commercial PHBV with processed PHBV and composites, as well as the related samples after injection moulding.

The results reported in Table 3 further suggest that the presence of the residue does not affect macromolecular weight loss, which is instead strongly influenced by thermal treatments. Indeed, even the injection moulding carried out at 165 °C, contributes to a further decrease of the macromolecular weight, confirming that PHBV tends to suffer from thermolysis. The following work was therefore focused on the composites prepared at the lower temperature and for a longer time, which was less affected by macromolecular weight loss, as well as proving greener and cheaper in terms of energy saving.

**Figure 7:**
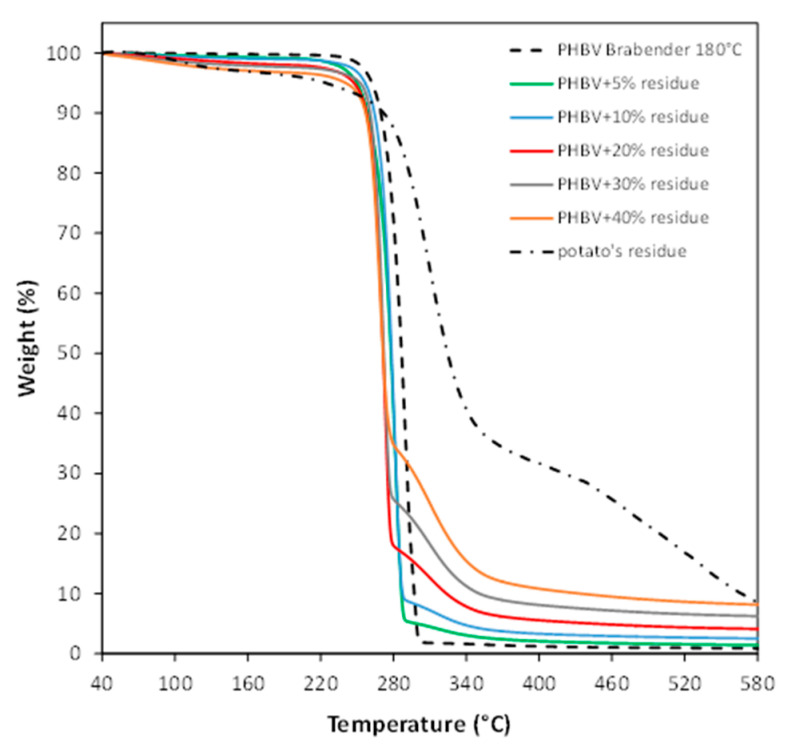
TGA curves of the sweet potato residue, PHBV processed at 180 °C and relative composites.

The thermal characterizations of the reference material and bio-composites are reported in Table 4, while Figure 7 shows the TGA curves of the polymeric samples. It is evident that PHBV degradation is realized in a single step and that the weight loss is completed just above 300 °C. Indeed, the accepted mechanism for PHB degradation consists of random breakage of ester bonds to vinyl ester and carboxyl groups through a single step [23]. On the other hand, all the bio-composites display a second degradation stage starting at about 280 °C, due to the additive decomposition. It is obvious that the filler content influences the entity of this step. Even the char residue is directly correlated to the filler amount: the higher the sweet potato content, the greater the amount of filler not eliminated by thermal degradations. It is also worth noting that the presence of the filler decreases both the initial decomposition temperature (*T*_onset_) and the maximum degradation rate (*T*_D_) of the PHBV composites: the effect becomes gradually more evident from the sample containing 10 wt % of filler onwards. In any case, it seems clear that the residue does not promote any degradation reactions, as indicated also by the GPC data reported in Table 3, which shows how the macromolecular weight reduction is independent of the residue amount.

PHBV is a semi-crystalline polymer characterized by a high level of crystallinity, as confirmed by the DSC data (Table 4) showing that the melting enthalpy is particularly high in both the first and the second heating scans (Δ*H*_m_: 79 and 82 J/g, respectively). Moreover, the polymer exhibits a high crystallization capability and crystallizes completely during the cooling step, while the glass transition of PHBV occurs at around 7 °C. 

**Figure 8:**
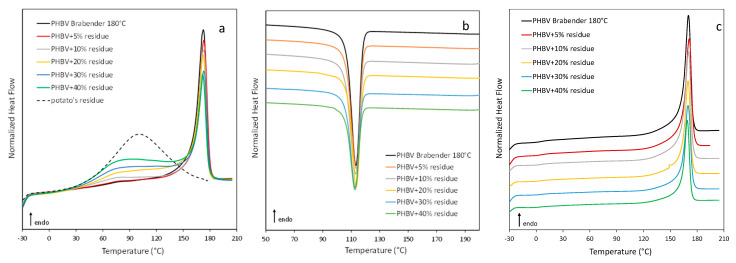
DSC curves of PHBV processed at 180 °C and relative composites (**a**) first scan, (**b**) cooling scan, (**c**) second scan.

As far as biocomposites are concerned, DSC curves, related to the first scan, show a progressively important endothermic process in the 25–60 °C temperature range (Figure 8) that can be ascribable to the enthalpy recovery due to the structural relaxation of the rigid amorphous fraction (RAF). This process disappears in the second scan. The endothermic process is notably more pronounced in the sample richest in residue, confirming that the filler induces constraints and impediments in polymer mobility, causing a larger amount of rigid amorphous fraction at the polymer/filler interface [60,61].

Moreover, Table 4 shows that in composites melting and crystallization temperatures do not change with respect to PHBV, suggesting that the crystal thickness is unchanged due to the presence of fillers and the sweet potato residue does not act as a nucleating agent for the polymeric matrix. 

The related enthalpies, calculated with respect to the PHBV content, do not display significant variations as a function of the filler amount. The crystalline weight fraction (*w*_c_) has been calculated by dividing the enthalpy of fusion of the first and second scans by the enthalpy of fusion of the 100% crystalline PHBV (ΔH_m_ = 143 J/g) [62]. Notably, *w*_c_ does not vary, confirming that the filler is not a nucleating agent for PHBV.

**Figure 9:**
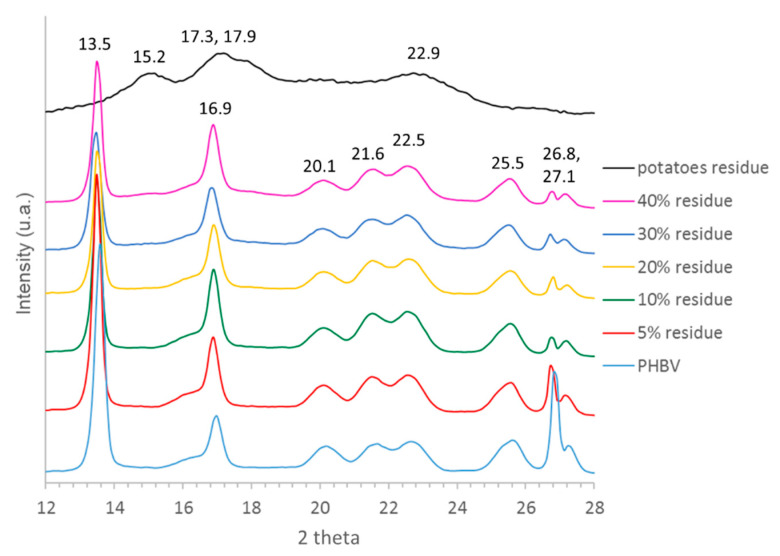
X-ray diffractograms of the sweet potato residue, polymeric matrix and related bio-composites.

The presence of the crystalline phase of the PHBV matrix has been confirmed by the X-ray diffraction analyses carried out on the films (Figure 9), whereas the starch crystal phase can be recognized only in the sample with the highest amount of sweet potato residue. Since the starch struggles to crystallize, even in the sample containing the highest residue amount, it can be speculated that the filler is well dispersed into the PHBV matrix. Moreover, it is notable that the WAXD traces of PHBV are not modified by the presence of the filler, even at high residue content. Only the decrement in the intensity of a few reflections (13.5° and 26.9°) is detected with the increment of filler quantity. These changes indicate that the original PHBV crystal structure is disturbed by the starch presence, probably due to the hydrogen bond interactions between PHBV and starch [63].

**Figure 10:**
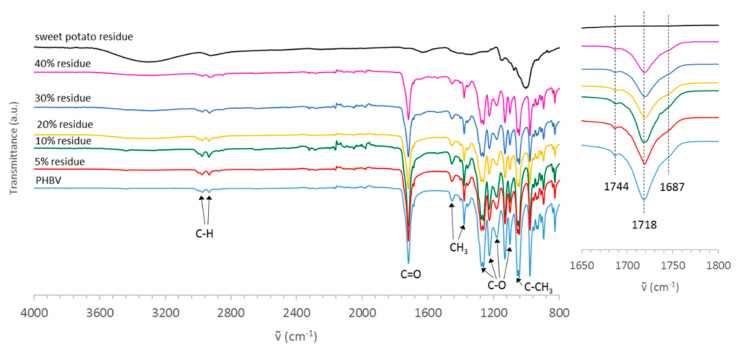
FT-IR spectra of the sweet potato residue, polymeric matrix and related bio-composites.

The presence of a certain degree of hydrogen bonds is confirmed by IR analyses carried out on the composites films and displayed in Figure 10.

The bands due to PHBV are clearly visible for all the blends and are prevailing on the starch bands, probably because the starch fraction is completely embedded into the PHBV matrix (film thickness: 300 µm) but the ATR-FTIR technique investigates the surface, penetrating only 1 µm deep. Therefore, the blends exhibit the IR bands due only to the PHBV component: stretching bands are identifiable at 2974 and 2932 for C–H and 1718 cm^−1^ for the carbonyl group in the crystalline fraction. Moreover, CH_3_ bending at 1450 and 1380 as well as C–O stretching at 1271, 1260, 1223, 1175, 1098 cm^−1^ can be found. Finally, the band of C–CH_3_ stretching at 1045 cm^−1^ is visible. However, the starch presence and particularly its interaction with PHBV through hydrogen bonds is perceptible in the change of carbonyl band, as shown in the magnification of Figure 10. Indeed, as already described by Zhang for starch/PHB blends [63], the hydrogen bonds between polyester carbonyl groups and starch hydroxyl functionalities involve a broadening of bands in the stretching carbonyl region. In particular, in the presence of hydrogen bonds, the band at 1718 cm^−1^ becomes less intense with a gradual relative increment of the shoulder around 1744 cm^−1^, due to the amorphous carbonyl group. At the same time, the band due to vibration of hydrogen-bonded carbonyls, centred at 1687 cm^−1^, tends to increase if compared with the main band at 1718 cm^−1^. 

**Figure 11:**
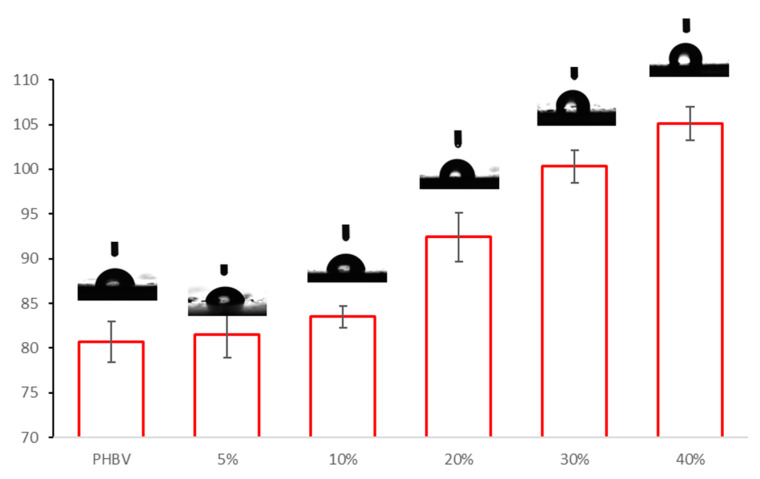
Water contact angle pictures for PHBV and all the bio-composites.

The presence of a certain amount of hydrogen bonds particularly involving the surface OH groups is further confirmed by the determination of water contact angle (WCA). Indeed, the addition of sweet potato residue to PHBV causes an increment of the WCA from 80.7 °C to 105.1° for the sample containing 40 wt % of sweet potato residue, as shown in Figure 11. This trend is unexpected since the main components of the sweet potato residue, i.e., starch and cellulose, are hydrophilic and water sensitive, whereas PHBV is a more hydrophobic material. However, this behaviour can be explained considering that starch form hydrogen interactions with carbonyl groups of the PHBV matrix, thus reducing the availability of hydroxyl ones, which are functionalities fit to interact with water through hydrogen bonds [64,65,66]. Moreover, other authors have reported that starch can effectively interact with PHBV as proven by the increment of storage modulus measured through dynamic mechanical analysis [67].

However, the described interactions do not suffice to create a good adhesion between the phases, as further evidenced by SEM analyses.

**Figure 12:**
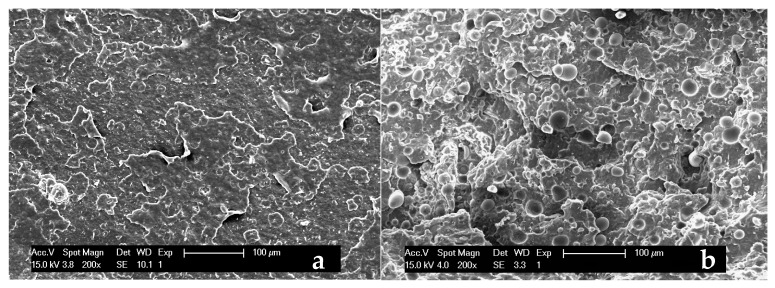
SEM pictures of fracture surfaces of (**a**) PHBV and (**b**) its biocomposite containing 20 wt% of sweet potato residue, at magnification 200×.

Figure 12a,b shows the microstructure of the fractured surface in both the raw PHBV and in its composite containing 20 wt % of sweet potato residue. While PHBV exhibits a roughly smooth aspect (Figure 12a), the starch granules are clearly recognizable (Figure 12b) in the polymer matrix of the composite, since they maintain their original morphology (Figure 4), undamaged by the mechanical and thermal stresses applied during the mixing and shaping processes. Consequently, the inner morphology described by Jagadeesan [68], consisting of smaller inside blocklets is not visible. The SEM analysis confirms that almost all the starch granules are also homogeneously dispersed in the matrix, even at the highest amount, not shown for sake of brevity, without any cluster formation. They also appear to be loosely bound to the matrix.

**Figure 13:**
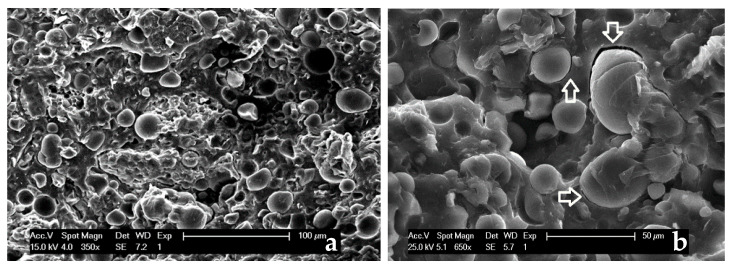
SEM picture of the biocomposite containing 20 wt% of sweet potato residue, (**a**) magnification 350×, (**b**) magnification 650×.

Only a few starch granules are still adherent to the flat-shaped phase, as can be seen in the central part of Figure 13a. The adhesion to the matrix is limited, as evidenced by the arrows in Figure 13b. Therefore, the SEM results suggest that the sweet potato residues can be successfully mixed with the polymeric matrix, but the phase compatibility is very limited.

**Figure 14:**
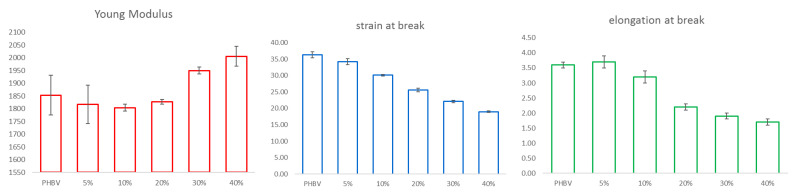
Tensile tests for composites prepared at 180 °C.

As to tensile properties, the data reported in Table 5 and depicted in Figure 14 show that Young’s modulus (E) is rather constant until 20 wt % of filler loading then tends to slightly increase with the increment of sweet potato residue. This behaviour is quite common [21,30,69,70] and can be ascribed to a weak interphase adhesion. At the same time, the tensile strength (σ) and the elongation at break (ε) decrease, confirming a poor impregnation between the additive and the matrix, as already demonstrated by the previous analyses.

## 4. Conclusions

The solid residue remaining after the starch extraction from Chinese sweet potatoes was used as an additive for the preparation of bio-composites based on PHBV. The resulting bio-composites are therefore completely bio-sourced and potentially biodegradable. They were prepared by melt mixing the polymer and an amount of sweet potato residue varying from 5 to 40% by weight. The composites are semicrystalline materials thermally stable up to 260 °C. A good dispersion of the additive into the bio-polymeric matrix was detected, probably due to a certain amount of hydrogen bond interactions between the phases, even if a poor interfacial adhesion was revealed by SEM, which in turn affected mechanical properties. However, the loss in mechanical properties is not so significant. Furthermore, the increased surface hydrophobicity opens a door to new application sectors for these biocomposite materials, which can satisfy the circular economy requests. Therefore, the present work contributes to enlarging the family of agro-industrial residues that can be valorized in the industry of new biomaterials with the aim to reach the zero-waste goal.

## Figures and Tables

**Table 1:** Composition of sweet potato residue (g/100 g, dry weight) *.

Component	Amount (wt %)
Starch	61.56 ± 0.72
Protein	3.81 ± 0.23
Fat	1.16 ± 0.01
Total dietary fibre	25.63 ± 0.17
Ash	1.95 ± 0.13

* about 6 wt % of moisture was detected

**Table 2:** Pectin, hemicellulose, cellulose and lignin content in sweet potato dietary fibre (%)

Pectin	Hemicellulose	Cellulose	Lignin
24.13 ± 0.79	13.66 ± 0.58	38.84 ±1.02	22.74 ± 0.69

**Table 3:** Gel permeation chromatography (GPC) results for different poly(3-hydroxybutyrate-co-3-hydroxyvalerate) (PHBV) samples.

Residue Amount (%)	Mixing Temperature (°C)	Processed by Injection Moulding	Mn · 10^−3^	Mw · 10^−3^	D
-	-	no	126.1	306	2.4
-	200	no	62.8	129	2.0
20	200	no	63.0	133	2.1
-	180	no	89.8	202	2.2
-	180	yes	47.8	122	2.6
5	180	no	90.7	217	2.4
5	180	yes	65.2	146	2.2
10	180	no	87.3	210	2.4
10	180	yes	51.1	113	2.2
20	180	no	88.7	217	2.4
20	180	yes	48.5	111	2.3

**Table 4:** Chemical and thermal characterization of composites prepared by melt mixing at 180 °C.

Residue Amount (wt %)	DSC									TGA		GPC		
	1st Scan			Cooling		2nd Scan				*T*_onset_ (°C)	*T*_D_ (°C)	*M*_n_ ·10^−3^	*M*_w_ ·10^−3^	*D*
	*T*_m_ (°C)	Δ*H*_m_ (J/g)	*w*_c_	*T*c (°C)	Δ*H*_c_ (J/g)	*T*_g_ (°C)	*T*_m_ (°C)	Δ*H*_m_ (J/g)	*w*_c_					
-	175	79	0.55	113	72	7	171	82	0.57	276	290	89.8	202	2.2
5	174	83	0.58	113	74	7	172	82	0.57	268	281	90.7	217	2.4
10	174	90	0.63	113	76	7	171	82	0.57	270	282	87.3	210	2.4
20	172	80	0.56	113	75	7	170	83	0.58	264	273	88.7	217	2.4
30	174	83	0.58	113	74	7	171	86	0.60	263	272	86.3	209	2.4
40	173	73	0.51	113	75	6	170	82	0.57	260	269	87.2	205	2.4

**Table 5:** Tensile data for composites prepared at 180 °C.

Residue Amount (wt %)	Young Modulus (MPa)	Tensile Strength (MPa)	Strain at Break (%)
-	1853 ± 78	36.3 ± 0.9	3.6 ± 0.1
5	1817 ± 76	34.2 ± 0.9	3.7 ± 0.2
10	1804 ± 14	30.1 ± 0.2	3.2 ± 0.2
20	1827 ± 9	25.6 ± 0.5	2.2 ± 0.1
30	1950 ± 14	22.1 ± 0.4	1.9 ± 0.1
40	2006 ± 39	19.0 ± 0.3	1.7 ± 0.1

## Data Availability

The data presented in this study are available on request from the corresponding author.
